# Neoantigen: A Promising Target for the Immunotherapy of Colorectal Cancer

**DOI:** 10.1155/2022/8270305

**Published:** 2022-02-15

**Authors:** Yue Zheng, Yang Fu, Pei-Pei Wang, Zhen-Yu Ding

**Affiliations:** Department of Biotherapy, Cancer Center, West China Hospital, West China Medical School, Sichuan University, Chengdu, China

## Abstract

At present, there are various treatment strategies for colorectal cancer, including surgery, chemotherapy, radiotherapy, and targeted therapy. In recent years, with the continuous development of immunotherapy, immune checkpoint inhibitors (ICIs) can significantly improve the treatment of advanced colorectal cancer patients with high levels of microsatellite instability. In addition to ICIs, neoantigens, as a class of tumor-specific antigens (TSA), are regarded as new immunotherapy targets for many cancer species and are being explored for antitumor therapy. Immunotherapy strategies based on neoantigens include tumor vaccines and adoptive cell therapy (ACT). These methods aim to eliminate tumor cells by enhancing the immune response of host T-cells to neoantigens. In addition, for MSS colorectal cancer, such “cold tumors” with low mutation rates and stable microsatellites are not sensitive to ICIs, whereas neoantigens could provide a promising immunotherapeutic avenue. In this review, we summarized the current status of colorectal cancer neoantigen prediction and current clinical trials of neoantigens and discussed the difficulties and limitations of neoantigens-based therapies for the treatment of CRC.

## 1. Introduction

Colorectal cancer (CRC) is the third most diagnosed tumor in the world, accounting for 10% of the total number of cases, and is the second leading cause (9.4%) of cancer-related death [1]. At present, the treatment strategies of CRC include surgery, radiotherapy, chemotherapy, and targeted therapy. Immune checkpoint inhibitors, including nivolumab and pembrolizumab, for programmed death receptor (PD-1) blockade have been approved for the treatment of CRC with deficient mismatch repair (dMMR) or high microsatellite instability (MSI-H) [2, 3]. Based upon the Phase III KEYNOTE-177 study (NCT02563002), pembrolizumab is currently approved for the first-line treatment of MSI-H/dMMR metastatic colorectal cancer [4]. However, immunotherapy with the anti-PD-1 monoclonal antibody did not achieve the desired effect for patients with proficient mismatch repair (pMMR) or microsatellite stability (MSS) [5, 6].

This phenomenon may be related to the fact that colorectal cancer with MSI-H usually has a higher tumor mutation burden (TMB). Due to more frequent insertion and deletion mutations in the DNA sequency and the frameshift mutations of opening read frame, solid tumors with MSI-H may produce and express more neotumor-specific peptides which are completely different from the self [7, 8]. Tumor-specific peptides are defined as neoantigens when they displayed by the major histocompatibility complex (MHC) on the surface of tumor cells, which can trigger a T-cell-mediated cytotoxic anti-tumor immune response and cause T-cell population expansion [9, 10]. These neoantigens are produced by cancer cells bearing mutations that affect the protein sequence and include nonsynonymous point mutations, codon insertion/deletion, frameshift mutations, splicing mutations, and gene fusions. There is also a subgroup of tumor-specific antigens (TSAs) that are derived from viral proteins [11].

The occurrence/progression of tumors is often driven by a series of somatic mutations across the genome. Mutations in genes that regulate cell division and growth, such as protooncogenes and tumor suppressor genes, are significant in the process of tumor formation [12, 13]. Moreover, the type and number of mutations can vary greatly between different tissues and tumor types, which means that neoantigens can be tumor-type-specific and even individual-specific. Patients with a higher tumor mutation load (TMB) will likely produce a greater number/variety of mutant peptides, which results in a greater abundance and variety of antigens. The mutation frequency of CRC is usually very high compared to most other cancer types, and the average mutation density is ~10 mutations per megabase [9].

Moreover, ~15% of CRC patients have microsatellite instability caused by defects in mismatch repair proteins, which is a hypermutable phenotype. This phenotype is most often associated with Lynch syndrome and hereditary nonpolyposis colorectal cancer (HNPCC) that meet Amsterdam II standards [14–18]. Hypermutable/hypervariable phenotypes with insertion/deletions (INDELs) in these short repetitive DNA sequences can lead to the formation of neopeptides if the mutations occur in coding regions. Some INDELs located within open reading frames are also frameshift mutations, which can lead to the production of vastly different proteins that contain large sections of miscellaneous amino acid sequences (depending on the codon sequence). Ultimately, this means that cancers with a hypermutable phenotype are more likely to produce a class of neoantigen that are associated with higher immunogenicity [7].

The neopeptides that are produced through the aforementioned means are expressed on tumor cells, whereas healthy cells will not present such antigens. The tumor-specific nature of neoantigens makes them ideal targets for antitumor immunotherapy and has been investigated for the treatment of CRC in a variety of basic and clinical immunotherapy studies. Nevertheless, the application of neoantigens for clinical immunotherapy is also faced with various challenges. Hence, this article reviews neoantigen technology, its prospects, and the challenges associated with the application of neoantigens for colorectal cancer therapy.

## 2. Identification and Selection of Candidate Neoantigens

**Figure 1:**
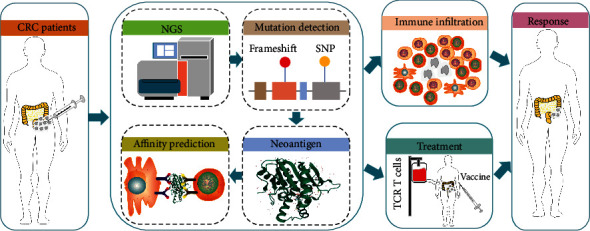
A brief schematic overview of prediction and the application of neoantigen and the application of neoantigen-based vaccines and ACT. Collect biological materials (tumor tissues) from patients with colorectal cancer. Predict, identify, and screen tumor neoantigens. The selected neoantigens are made into vaccines, or corresponding T-cells are expanded for patient treatment. Neoantigen vaccines or ACT can be used in combination with adjuvants, chemotherapy, radiotherapy, and/or immune checkpoint inhibitor.

Neoantigens need to fulfill two criteria to be useful as the target of immunotherapies, namely, they need to be (1) presented by MHC molecules and (2) induce a CD4+ or CD8+ T-cell immune response. Thus, predicting/identifying neoantigens that can be explored in clinical research is the starting point for exploiting them as targets for tumor immunotherapy. This can be a complex and comprehensive process with three steps: (1) the identification of somatic mutations in DNA or mRNA sequences and the neopeptides produced from those mutations, (2) an assessment of the neopeptide binding affinity and capacity for presentation by MHC I/II molecules [19], and (3) the determination of whether the neoepitopes can induce T-cell proliferation and their related immune responses. It is notable that different prediction pipelines/algorithms can vary substantially due to features such as the treatment schedule, economic circumstances, and technical limitations [8, 20, 21]. We described the brief flow of neoantigen prediction and the use of neoantigen in the treatment of CRC in Figure 1.

At present, the development of high-throughput sequencing (second-generation sequencing, NGS) technology has increased the feasibility for the accurate identification of mutations using both DNA and RNA. This requires comparing tumor (somatic) whole-genome (WGS) or whole-exon sequencing (WES) data to that from healthy (germline) tissue [19]. Moreover, RNA-sequencing can help infer the expression and therefore viability of mutant peptides with tumors. The sequencing data can also have utility for the identification of HLA genotypes (HLA allele detection), which is an important aspect that can be used to supplement the analysis when determining the neopeptide presentation and binding affinity in later stages of the pipeline [22–24].

Several computational approaches based on machine learning (ML) algorithms that account for HLA alleles have been used to predict neopeptide binding affinity and processing [25, 26]. Such methods are based on a large number of experimental data sets that detail HLA binding. Additionally, immunopeptideomic approaches that utilize state of the art mass spectrometry (MS) analyses can directly assess neopeptides that are displayed by class I and class II MHC molecules, which can be useful for the verification of the results that were predicted via in silico analyses [27–30]. For example, NetMHCpan and NetMHCIIpan can integrate information form in silico and MS analyses to predict the presentation of neopeptides and their HLA binding affinity [31–34]. There are also computational pipelines, such as pVACtools and MuPeXi, which predict and priorities neopeptides by integrating WGS, mutant cloning, mRNA expression, peptides processing, and HLA binding affinity data [24, 35–38].

A major difficulty for the application of neoantigens as immunotherapy targets is the need to predict whether the neoantigens will be recognized by T-cell receptors (TCRs) and stimulate T-cell activation and infiltration. There are a number of T-cell-based assays that can be used to measure tumor-related T-cell responses, including enzyme-linked immunosorbent spot (ELISpot) detection [39] and multicolor tetramer-based flow cytometry [40]. Peng et al. reported that nanoparticle- (NP-) barcoded nucleic acid cell sorting (NACS) can be used to enumerate and isolate neoantigen-specific CD8 + T-cells. Additionally, a mutation-related neoantigen-specific function extension (MANAFEST) analysis has the ability to sensitively monitor neoantigen-related antitumor immune responses via the molecular characterization of related TCR sequences [41].

A global community with researchers from many institutions has established a Tumor Neoantigen Selection Alliance (TESLA). Different teams in the alliance independently mine shared data, predict potential neoantigens, and prioritize candidate neoantigens. The results from each team are crossmatched and combined, with the aim to understand the immunogenicity of tumor epitopes and improve neoantigen prediction algorithms [42]. For example, Bai et al. proposed the “NP” rule based on the conservative mutation direction of anchor residues from immunogenic neoantigens and integrated the rule within existing prediction algorithms to improve neoantigen immunogenicity prediction [43].

## 3. Neoantigens and Immune Response in Colorectal Cancer

The median TMB of colorectal cancer ranked seventh among 30 of the most common types of malignant tumors. About 16% of CRCs have a TMB of >12 mutations per 106 base pairs, which are classified as highly mutated tumors [44]. Patients with higher TMB may present more candidate neoantigens that can be used for clinical treatment. However, the type of mutation can also have a great impact upon the clinical applicability of the neoantigen. We list the mutated antigens that were found or studied in CRC in Table 1.

### 3.1. Frameshift Peptide

For MSI-H CRC, frameshift mutations caused by INDELs can lead to the production of new frameshift peptides (FSP), which are the main source of neoantigens in these tumors [17]. Frameshift protein sequences represent a novel tumor-specific antigen subclass that can induce FSP-specific immune response [45]. Frameshift mutations can be commonly found in genes with important biological functions in most MSI-H colorectal tumors. These genes have functions including epigenetic regulation (HDAC2, ARID1A), DNA repair (MSH3 and MSH6), signal transduction (TGF*β*RII, IGFR2, ACVR2A), cell apoptosis (BAX), and miRNA processing (TARBP2, XPO5). Moreover, there is a correlation between the density of CD8+ tumor infiltrating T lymphocytes and the number of mutations [46–49]. Tumor growth factor *β* receptor II (TGF*β*RII) mutations are commonly found in MSI-H CRC (90% of HNPCC) but can also be present in ~15% of MSS CRC [17]. The short peptide produced by a frameshift mutation within TGF*β*RII can cause the proliferation of CD4 + T-cells in the tumor infiltrating lymphocyte (TIL) population [7, 50]. This is associated with the specific killing of cells bearing this mutation in an HLA-A2-restricted manner, which makes it a prime target for tumor vaccine therapy [7]. Indeed, Inderberg et al. have reported that the immunogenic neopeptide produced by a -1A mutation within the TGF*β*RII microsatellite A [10] tract can induce the HLA-A2-restricted TGF*β*RII mutation-specific T-cell immune response and increase survival in a CRC mouse model [51].

A number of other promising neoantigens arising from frameshift mutations have also been described that can cause related cytotoxic T lymphocyte antitumor responses, such as the HLA-A0201-restricted neoantigen caused by frameshift mutations within O-linked N-acetylglucosamine transferase (OGT) and the -1A mutation within the A [8] tract of MSH3 [47, 52]. It has been reported that tumor-specific peptides produced by frameshift mutations in the coding region of CDX2 in patients with colorectal cancer can cause relative antibody immune responses in serum [53]. Speetjens et al. reported immunogenicity tests for 15 peptides with microsatellite frameshift mutations, of which 8 antigens (TGF*β*RII-1, MARCKS-1, MARCKS-2, CDX2-2, BAX*α*+1, PCNXL2-2, TCF7L2-2, and TAF1B-1) can be combined with MHC molecules and presented to T-cells (the four foremost antigens being the most significant) [54].

### 3.2. Single-Nucleotide Variants and Shared Mutation Peptides

In addition to specific neoantigens produced by individual mutations, driver gene mutations in genes such as KRAS, TP53, and BRAF can be commonly found in multiple tumor types [55, 56]. Point mutations and single amino acid substitutions in KRAS can cause its activation and affect cell proliferation, division, and apoptosis via the intracellular signaling cascade in about 40% of CRC patients [57–60]. The most frequent KRAS mutation is located in its second exon (most commonly resulting in G12D, G12V, and G13D) [60, 61]. It has been reported that peptides produced by high-frequency point mutations in KRAS can stimulate the proliferation of cytotoxic T lymphocytes (CTLs) in vitro and in CRC patients [61–63]. Iiizumi et al. found that mutant peptides produced by driver gene mutations are immunogenic, including KRAS-G12D, KRAS-G12R, KRAS-G13D, and PIK3CA-H1047R. And the results show the neoantigens that stimulate the response of CD4+ T-cells (rather than CD8+), which indicate that a greater number of the neoantigens are HLA-II-restricted [64]. This phenomenon has also been previously reported [65]. For TP53, the most commonly mutant gene in multiple tumors, Lo et al. found that TP53 p.R175H can trigger a TCR-mediated immunogenic response, and that the immunogenicity was HLA-A^∗^0201-restricted. This feature has also been recognized in ovarian cancer, uterine cancer, and myeloma cell lines. Transduction with a retrovirus encoding HLA-A^∗^0201 can also cause the recognition of colorectal cancer cells with TP53 p.R175H [66]. In a humanized mouse colorectal cancer model, a vaccine produced by using a mixture of long peptides derived from KRAS and TP53 mutant proteins combined with MHC molecules was found to induce strong cytotoxicity and T helper cell immune responses to multiple mutations at the same time [67]. Recently, it has been reported that single-nucleotide variants (SNVs) in genes, such as KRAS, PIK3CA, PCBP1, and CHEK2, are associated with the production of the 10 most frequent neoantigens. An analysis of the published CRC WES data has enabled a more complete map of CRC mutations to be produced, which indicated that high-frequency mutations such as KRAS G12D, KRAS G12V, PIK3CA E545K, PIK3CA H1047R, and BMPR2 N583Tfs _∗_ 44 can combine with HLA and be presented [68]. However, recent findings indicate that this selection of mutated driver genes may have weak binding affinity with MHC molecules during the development of tumors.

### 3.3. Microsatellite Stability

The neoantigens mentioned above all appear in MSI-H CRC, which is associated with a higher tumor mutation burden. However, most CRCs have relatively lower mutation burden (MMR-p/MSS CRC) and do not benefit from treatment with immune checkpoint inhibitors [6].

Nevertheless, studies have focused upon the possibility and potential for neoantigen immunotherapy in CRC patients with a low TMB. Ovarian cancer, glioblastoma, metastatic cholangiocarcinoma, and other tumors with low TMB have been reported to have positive effects following treatment with personalized vaccines against neoantigens and adoptive cell transfer (ACT) therapy with reactive T-cells [69–73]. CRC patients with a low TMB are affected less by immune escape events (such as antigen presentation defects) and may benefit from neoantigen vaccines or ACT. Bulk et al. investigated the autologous neoantigen-specific T-cell immune response of patients with MSS CRC. The results showed that there were specific T-cell immune responses against multiple neoepitopes in three patients, and the existence of neoantigen-specific T-cells in the CD39 + CD103 + T-cell subset was confirmed by TIL sorting [74]. The study of Tran et al. confirmed that TILs can be used to detect neoantigen-directed T-cell reactivity in gastrointestinal tumors (including those with moderate mutation burden) [75]. In addition, studies have found that there are immune responses to new epitopes by TILs from metastatic tumors (including MSS CRC) [76].

## 4. Clinical Trial of Neoantigens as Targets in Colorectal Cancer

### 4.1. Neoantigen Vaccine

The phenomenon of eliciting effective neoantigen-specific antitumor T-cell immune responses and inhibiting tumor growth has been observed in vivo. Such preclinical experiments have highlighted the potential of neoantigen-based immunotherapy as a new therapeutic strategy. The efficacy of neoantigen vaccines has also been confirmed in the mouse models of CRC [30, 65]. Clinical trials of neoantigen vaccines have been conducted for melanoma and glioblastoma, which proved that the vaccine is a safe method for eliciting tumor-specific T-cell responses [71, 77–79]. Moreover, there are currently several clinical trials exploring the efficacy and safety of vaccines against different kinds of neoantigens for CRC patients (Table 2).

A pilot study targeting a KRAS mutant peptide demonstrated that only two of the seven CRC patients showed a positive immune response after vaccination [80]. Kloor et al. recently conducted a phase I clinical trial to evaluate the safety and immunogenicity of frameshift peptide neoantigen-based vaccines for dMMR CRC patients. The trail used a vaccine based on FSP neoantigens derived caused by AIM2, HT001, and TAF1B mutations. Their results showed that humoral and cellular immune responses were induced by at least one of the frameshift peptide vaccines for all 22 of the dMMR CRC patients, and there were no serious vaccine-related adverse reactions [81].

Attempts to enhance the immune response generated against neoantigen vaccines through the use of GM-CFS as an adjuvant were successful. However, the vaccine was not found to be beneficial for patient's disease progression, which may be related to an increase of immune regulatory cells [82]. In addition, many neoantigen vaccines have been tested in preclinical mouse models. Ni et al. developed a neoantigen (Adpgk) nanovaccine (banNV) with a Toll-like receptor 7/8 agonist R848 and TLR9 agonist CpG as a dual adjuvant. It was found that the dual adjuvant neoantigen vaccine increased the immunogenicity of the neoantigen and elicited a good antitumor response together with anti-PD-1 therapy [83]. It has also been reported that the use of multiple neoantigen DNA vaccines and anti-PD-1 therapy can synergistically control the growth of the MC38 colon cancer cell line [84]. Kim et al. reported that the combination of a neoantigen-based EpiGVAX vaccine and 5-aza-2′-deoxycytidine can increase the antitumor efficacy of an irradiated whole-cell CRC vaccine by inducing neoantigen-specific antitumor T-cell response [85]. Leoni et al. recently selected 209 shared FSPs in the MSI CRC genome map database to produce a viral vector vaccine, Nous-209, which was confirmed to activate human CD8+ T-cells via in vitro experiments [86].

### 4.2. Adoptive Cell Transfer

ACT is a type of immunotherapy that transfers immune cells to patients. T-cells that specifically recognize neoantigens and are capable of inducing antitumor response can be ideal carriers for ACT. Inderberg et al. used T-cells transduced with HLA-A2-restricted TGF*β*RII mutation-specific TCR in a mouse colorectal cancer model and found that it reduced tumor growth and improved survival [51]. A clinical trials reported that the autologous transfer of a HLA-C^∗^08 : 02-restricted KRAS G12D reactive polyclonal CD8+ T-cell population successfully treated patients with metastatic CRC [87]. Tran et al. expanded KRAS G12D-specific CD8+ T-cells isolated from the lung metastasis in CRC patients, and the subsequent infusion caused the metastases to complete resolve for six out of the seven patients [88]. These studies suggest the potential of neoantigen-specific T-cells in the treatment of CRC, and other clinical trials of ACT based on neoantigens are currently underway (Table 2).

## 5. Challenges and Conclusion

Studies have proven that MSI-H CRC patients have a higher survival rate and therapeutic effectiveness following immune checkpoint inhibitor therapy [5, 6, 89, 90]. The neoantigens produced by gene mutations can cause antitumor immune responses, and the specific T-cells that recognize neoantigens are not affected by thymus central tolerance [91]. This feature makes neoantigen-based vaccines, ACT, and other immune-based treatments a promising strategy for the treatment of colorectal cancer. These treatments may even be used to prevent cancer formation in tumor-free Lynch syndrome mutation carriers. MSS patients with a low TMB are currently considered unsuitable for immunotherapy strategies. However, some studies have found that there are still more than one neoantigen (related to the CRC molecular subtype) that can produce an immune response in such patients [76]. Neoantigen-related immunotherapy strategies have also achieved a good level immunoreactivity in other tumors with low TMB, such as glioblastoma.

Most neoantigens are patient-specific, and the process for clinical testing of individual neoantigens in patients is limited by prediction technology, economic costs, and other aspects. Moreover, tumor sequencing generally only reveals the mutations of some cells within the tumor; hence, intratumor heterogeneity usually is not considered. Heterogeneity between the primary lesion and the metastasis may also hinder the neoantigen based therapies. Tumor heterogeneity as a mechanism of treatment failure and disease recurrence/progression should therefore be considered during the identification, selection, and clinical application of neoantigens.

Among patients with CRC, especially those with MSI-H phenotype, neopeptides derived from frameshift mutations account are predominant. However, the current neoantigen prediction technologies are mostly used for the prediction of neoantigens derived from SNVs; thus, the prediction of neoantigens generated by frameshift mutations remains problematic. It is notable that if the binding affinity of driver gene neoantigens (e.g., TP53 and KRAS) resulting hotspot mutations with MHC was improved, they could be used as therapeutic targets for a larger number of patients with a variety of cancer types, thus enabling more patients to receive immunotherapy.

The ability of a neoantigen to induce an adaptive immune response after binding to MHC molecules is affected by many factors, including the HLA presentation function of the antigen, mutations that regulate the HLA expression, peptide transport, and the characteristics of HLA itself.

Even though neoantigens can cause local or systemic increase in specific T-cells, the immune function is still affected by inhibitory factors in the immune microenvironment (such as Tregs and M2 macrophages). The use of chemotherapy, radiotherapy, and oncolytic viruses can enhance the inflammatory response and may be a promising method to support immunotherapy.

In short, neoantigens are a new immunotherapeutic strategy for treatment of various types of CRC. However, there are still many challenges in ranging from the clinical application to the neoantigen prediction/screening, which still need to be further explored.

## Figures and Tables

**Table 1:** List of mutated antigens that were found or studied in CRC.

Gene	Type of mutation	Epitope	HLA	Reference
OGT	Frameshift mutation	SLYKFSPFPL (FSP06)	HLA-A0201	[48]
TGF*β*RII	Frameshift mutation	p523, SLVRLSSCV p573, RLSSCVPVA p577, SSCVPVALM p578, LSSCVPVAL p579, VPVALMSAM p537, AMTTSSSQKNITPAILTCC p538, SLVRLSSCVPVALMSAMTTSSSQ p539, ALMSAMTTSSSQKNITPAILTCC p540, SPKCIMKEKKSLRLSSCVPVA p541, PKCIMKEKKKSLVRLSSCV p542, SPKCIMKEKKAW p543, PKCIMKEKKKAW p621, KSLVRLSSCVPVALMSAMT	—	[51], [52], [53]
Bax	Frameshift mutation	p517, RHPSWPWTRCLRMRPPRS p518, IQDRAGRMGGRHPSWPWTRCLR p519, GGTRAGPGPGASGCVHQEAERV p520, ASGCVHQEAERVSQAHRGRTGQ p521, IQDRAGRMGGGGTRAGPGPGAS	—	[51]
MSH3	Frameshift mutation	FLLALWECSL (FSP18) LLALWECSL (FSP19) IVSRTLLLV (FSP23) LIVSRTLLLV (FSP31)	HLA-A0201	[54]
CDX2	Frameshift mutation	—	—	[55]
FTO	Frameshift mutation	TLSPGWSAV	HLA-A0201	[56], [92]
Caspase 5	Frameshift mutation	FLIIWQNTM	HLA-A0201	[56], [93]
*KRAS*	SNVs	G12D, VVVGADGVGK G12V, VVGAVGVGK G12A, VVVGAAGVGK	HLA-A1101	[65], [70]
PIK3CA	SNVs	—	—	[70]
PARVA	SNVs	NLPLSPIPFELDREDTMLEENEVRT	—	[76]
G3BP1	SNVs	NCHTKIRHVDAHTTLNDGVVVQVMG IRHVDAHTTL	—	[76]
ACTR10	SNVs	SVPEGVLEDIKAHTCFVSDLKRGLK	—	[76]
RAE1	SNVs	WWLETLAQPELFLSTLPHLCTNLGP	—	[76]
PDP1	SNVs	PKSEAKSVVKQDWLLGLLMPFRAFG SEAKSVVKQDW SEAKSVVKQDWL	—	[76]
QRICH1	SNVs	VHVSGSPTALAAFKLEDDKEKMVGT	—	[76]

**Table 2:** Clinical trials of neoantigens in colorectal cancer.

Type of therapy	Study phase	Tumor	Status of CRC	Strategy	Combination therapy	Number of patients	Status	Trail number
Vaccine	Phase I	Pancreatic cancer metastatic Colorectal cancer metastatic	MMR-p	Neoantigen vaccine with poly-ICLC adjuvant	Retifanlimab	12	Not yet recruiting	NCT04799431
Vaccine	Phase I/II	Colorectal cancer	Germline MMR-d, MSI-positive	Vaccination with frameshift-derived neoantigen-loaded DC	—	25	Active, not recruiting	NCT01885702
Vaccine	Phase I	Gastric cancer Hepatocellular carcinoma Non-small-cell lung cancer Colorectal cancer	—	Neoantigen-primed DC cell vaccine	—	80	Recruiting	NCT04147078
Vaccine	Phase I/II	Non-small-cell lung cancer Colorectal cancer Gastroesophageal adenocarcinoma Urothelial carcinoma	MSS	GRT-C901/GRT-R902, a neoantigen cancer vaccine	Nivolumab and ipilimumab	214	Recruiting	NCT03639714
Vaccine	Phase I/II	Non-small-cell lung cancer Colorectal cancer Pancreatic cancer Solid tumor Shared neoantigen-positive solid tumors	MSS	GRT-C903 and GRT-R904, a shared neoantigen-based therapeutic cancer vaccine	Nivolumab and ipilimumab	144	Recruiting	NCT03953235
Vaccine	Phase I	Colorectal cancer Pancreatic cancer	MMR-p	Pooled mutant-KRAS peptide vaccine with poly-ICLC	Nivolumab and ipilimumab	30	Recruiting	NCT04117087
Vaccine	Not applicable	Advanced esophageal squamous carcinoma Gastric adenocarcinoma Pancreatic adenocarcinoma Colorectal adenocarcinoma	—	Personalized mRNA tumor vaccine encoding neoantigen	—	24	Recruiting	NCT03468244
Vaccine	Phase I	Colorectal adenocarcinoma Pancreatic ductal adenocarcinoma	—	Personalized synthetic tumor-associated peptide vaccine	Imiquimod, pembrolizumab	60	Recruiting	NCT02600949
Vaccine	Phase I	Colorectal cancer Breast cancer Head and neck squamous cell carcinoma Melanoma Non-small-cell lung cancer Pancreatic cancer Liver cancer	—	QUILT-2.025 NANT Neoepitope Yeast Vaccine (YE-NEO-001)	—	16	Recruiting	NCT03552718
Vaccine	Phase I/II	Melanoma Colon cancer Gastrointestinal cancer Genitourinary cancer Hepatocellular cancer	—	Messenger RNA- (mRNA-) based, personalized cancer vaccine against Neoantigens	—	5	Terminated (slow accrual)	NCT03480152
Adoptive T-cell therapy	Phase I	Solid tumor	—	Gene-edited autologous NeoTCR-T-cells (NeoTCR-P1)	Nivolumab or IL-2	148	Recruiting	NCT03970382
Adoptive T-cell therapy	Phase I	Cholangiocarcinoma Colorectal carcinoma, digestive system carcinoma Esophageal carcinoma Gastric carcinoma Pancreatic adenocarcinoma	—	CD8 + T-cells against personalized peptide antigens	Pembrolizumab	1	Terminated	NCT02757391
Adoptive T-cell therapy	Phase I/II	Colorectal cancer	MSI	TCR-engineered T-cells against TGFBRII frameshift peptide	—	1	Terminated	NCT03431311
